# Submucous Injection as a Cure for the Toothache of Pregnancy

**Published:** 1860-01

**Authors:** Horatio R. Storer


### ARTICLE XIX.

*Submucous Injection as a Cure for the Toothache of Pregnancy.* By HORATIO R. STORER, M. D.

IN a paper read before the society for medical observation in February last,* it was suggested that in certain obstetric cases, otherwise incurable, permanent relief might be had by a modification of the simple and easy process of Alexander Wood. As the remarks referred to were but incidentally made, in connection with a case illustrative of criminal abortion, and as the proposal itself seems to have been new to the profession, it may be worth repeating in more distinct form, and at greater length.

"Pains in the teeth," says Montgomery, "are with some the invariable accompaniment of pregnancy."† "Their effects upon the comfort and well being of the patient are often very distressing."‡ "After all our endeavors, we shall find ourselves in many instances unsuccessful," "and if not relieved, abortion may result.§

Not to enumerate the long list of remedies that have hitherto been advised for the malady, it is only necessary to state that in certain obstinate cases they are all found unavailing, and that general practice and many writers have been in favor of proceeding to the extraction of a tooth; if which fail, some have been rash enough to counsel the deliberate induction of abortion. These acts however, must be pronounced unjustifiable, both on moral and legal grounds, which I have elsewhere adverted to.||

*American Journal of the Medical Sciences, April, 1859, p. 314, copied by Atlanta Medical and Surgical Journal, &c.

†Signs and Symptoms of Pregnancy, 2d edit., p. 283.

‡Churchill, Diseases of Females, p. 338.

§Ibid., pp. 339, 340; Campbell, Midwifery, p. 519; Capuron, Mal. des Femmes, p. 357.

||North American Med. Chir. Review, May, 1859, p. 459; July, 1859, p. 657.

The toothache of pregnancy, as such, is not dependent upon caries, and if for scientific reasons alone it should not be submitted to the usual treatment. Decay may undoubtedly exist, and in pregnancy caries of the teeth often does progress much more rapidly than at other times, but the affection we are now considering being purely sympathetic and reflex in its nature, dependent directly upon the uterine irritation, can seldom be relieved by the extraction of the tooth, whether this be sound or impaired.

Burns, Blundell and others acknowledge that extraction of a tooth during pregnancy may be immediately followed by abortion—a consequence that might readily be expected from so profound an impression upon a nerve just then in unnatural and excessive sympathy with the uterus; in much the same manner as abortion sometimes occurs in consequence of intentional excitation of the mammæ, whose system of nerve is now allowed to be in intimate connection with the uterine. Be this as it may, however, from the fact of the occurrence and its risk—that abortion may ensue and frequently has ensued directly in consequence of the extraction of a tooth during pregnancy—we are compelled to assert that this very unscientific operation should always be avoided. To the argument that the pain, if unrelieved, might of itself occasion miscarriage, we should answer, even if we had no alternative to propose, that at times this neuralgic pain has suddenly and spontaneously ceased without interference, and that at others, however severely abortion may have threatened, it yet has not occurred.

But if the extraction of a tooth is to be reprehended in such cases; much more is the intentional extraction of the fœtus. The growth and moral tone of society, and the best interests of the profession, have already suffered too much at the hands of rash and meddlesome practitioners, who have thus abetted, however unintentionally, the spread of criminal abortion. We are plain in our statement, but unhesitating, for we believe that in much of the treatment of the nervous complications of pregnancy, whether evidenced by vomiting, convulsions, mania or simply toothache, there is oftentimes on the part of the attendant a carelessness resulting in direct intra-uterine murder—and that by the influence of such example upon the moral sense of the community, the frequency of the intentional crime is increased. The remark we have now made applies with equal force to much also of the usual treatment of difficult childbirth—to recklessness, by what authorties and however defended, in the use of the crotchet, the plug and of ergot.

In considering the real character and cause of the toothache of pregnancy, it occurred to my mind that the operation which has of late been found so effectual in removing neuralgic pains from other portions of the body, would probably prove equally valuable when applied to the gums, and in practice I have found it perfectly successful for this purpose.

CASE.—A. Z., aged 22, applied to me for treatment early in May last. Patient had suffered for several weeks from severe neuralgic pain throughout the left half of the upper jaw, at times lancinating in its character, at others more dull, but never wholly absent. The general health was decidedly affected, as evidenced by the state of the circulatory, digestive and nervous systems. The teeth on inspection, were all sound; there was no heat or swelling of the gums, no tenderness or increase of pain on pressing them.

Anodynes, both local and general, refrigerants, em[illegible]-ent poultices, and counter-irritants were successively [illegible]e-sorted to, without benefit. After much solicitation, a tooth was extracted; the patient remained unrelieved. On the following day, no change for the better having occurred, ten drops of the Edinburgh solution of the bimeconate of morphia were injected beneath the mucous membrane of the gum; the pain ceased instantaneously, and from that moment to the present, a period of nearly five months, there has been no return of the malady.

Should the operation prove insufficient entirely to arrest the pain, I should advise a direct attempt upon the organ causing the disturbance, by local applications to the cervix uteri. These do not seem to have been resorted to for this special purpose, but from the tincture of iodine we might expect the same benefit as in the vomiting of pregnancy; cases of which have lately been reported in this Journal by Dr. Miller, of Dorchester, the treatment referred to not being original with himself. Too much caution, however, cannot be used in meddling with the uterus during pregnancy; the employment of the speculum, often enough to be condemned at other times, usually becomes here doubly unjustifiable.

I am not aware that any writer has hitherto proposed to prevent, by submucous injection, the extraction of teeth during pregnancy—and thereby to prevent abortion, resulting either from that operation or from the neuralgic pain; not even does the induction of mere local anæsthesia, by any of the numberless modes attempted, seem to have been thought of for this special purpose. Nor do I know that submucous injection had ever been made use of previously to the case I have related, for the cure of dental neuralgia. It was the opinion of a friend, Dr. Page, at the meeting of the society to which the subject was originally presented, that upon this point I was in error; but the gentleman has subsequently informed me that the application in the cases to which he alluded, in the practice of a distinguished dentist, was of anodynes externally, to the surface of the gum. Apparently the only instances as yet recorded of the injection of opiates into the substance of the gum, are by a dentist of Edinburgh, Mr. Smith;* and the operation with him was for the purpose of producing temporary local anæsthesia during the extraction of teeth, not for the purpose of preventing their extraction by the cure of neuralgia.

By the copying of a large portion of my former paper

* Edinburgh Medical Journal, November, 1858, p 424

into a leading journal of American dentistry,* an unexpected opportunity has been given of impressing upon dentists the heavy responsibilities, hitherto generally unacknowledged by them, that attach to the extraction of teeth from pregnant women; and I cannot but hope that the views expressed may thus be made productive of decided and extensive good.

[*Boston Med. and Sur. Jour.*

*Dental Cosmos (new series of Dental News-Letter,) August, 1859, p. 53.

